# Patient perspective on psoriasis: Psychosocial burden of psoriasis and its management in Malaysia

**DOI:** 10.1371/journal.pone.0305870

**Published:** 2024-07-18

**Authors:** Azura Mohd Affandi, K. Thiruchelvam

**Affiliations:** 1 Consultant Dermatologist, Hospital Kuala Lumpur, Kuala Lumpur, Malaysia; 2 Medical Advisor of Psoriasis Association of Malaysia, Selangor Darul Ehsan, Malaysia; 3 Psoriasis Association of Malaysia, Selangor Darul Ehsan, Malaysia; Rajiv Gandhi University of Health Sciences, INDIA

## Abstract

**Background:**

Psoriasis is a chronic erythematous inflammatory skin disorder. The major challenge with psoriasis is delayed diagnosis, resulting in delayed treatment initiation and reduced quality of life (QoL).

**Objective:**

This patient perspective study aimed to explore the emotional and psychosocial burdens faced by patients with psoriasis in Malaysia and their attitudes toward current psoriasis treatment.

**Methods:**

Adult patients with mild or moderate-to-severe plaque psoriasis, preferably with concomitant psoriatic arthritis, participated in a patient advisory board meeting along with a senior consultant dermatologist. Patients had to describe their initial symptoms, time of diagnosis, misdiagnosis, treatment initiation delays, treatment course, flare-ups, psychosocial impact, and QoL associated with psoriasis.

**Results:**

The 11 participating patients had a mean age of 46 years with mean age of psoriasis diagnosis and an average year of suffering with psoriasis being 21.9 years and 24.5 years, respectively. The most common initial symptom of psoriasis was itching (62.5%), particularly of the scalp followed by itchiness and red patches on skin. Most patients (90%) reported initial misdiagnosis with other skin diseases by their primary care physicians (PCPs), which led to delayed treatment initiation. Most patients reported an emotional impact of psoriasis, including low self-esteem (18%), lack of confidence (27%), shock (18%), sadness (9%), and outrage (9%). Social discrimination/stigmatization in public places and at work (45%), and even from relatives (18%) was another reported challenge. However, 73% of patients were highly satisfied with the current treatment. Overall, the patients agreed that the lack of public awareness of psoriasis was responsible for the social stigma.

**Conclusions:**

The evidence obtained from this qualitative study indicated that psoriasis has a significant emotional and psychological impact on the patients affecting their QoL. Lack of awareness of the disease among PCPs, patients, and the public is a major challenge leading to poor treatment outcomes.

## Introduction

Psoriasis is a chronic inflammatory disorder of the skin, characterized by erythematous scaly plaques involving the elbows, limbs, trunk, scalp, and sacral region [1, 2]. Although it affects people of all ages, it is seen more in adults than in children [3, 4]. It is a serious global problem with the prevalence ranging from 0.09% to 11.4%, and approximately 100 million people are estimated to be affected globally [4]. Plaque psoriasis affects 93.4% of patients with psoriasis in Malaysia, with a male: female ratio of 1.3:1, and the mean age of onset is 35.39 ± 16.19 years [5].

Psoriasis shows varied dermatological manifestations based on which it is classified into different clinical types. Plaque psoriasis or psoriasis vulgaris is the most predominant type of cutaneous psoriasis. Other types of cutaneous psoriasis include guttate, inverse, pustular, and erythrodermic [1, 2]. The pathophysiology of psoriasis is diverse; both genetic and environmental factors may cause hyperproliferation and aberrant skin inflammation, contributing to the development of psoriasis [3, 6]. Derangements in immune responses (activation of different pro-inflammatory cytokines) are responsible for the inflammation associated with psoriasis [1, 7, 8].

Psoriasis is not limited to the skin; it is a systemic inflammatory disease affecting different organ systems. It is associated with various comorbidities such as psoriatic arthritis (PsA), diabetes mellitus, hypertension, hyperlipidemia, obesity, metabolic syndrome, cardiovascular disease (CVD), myocardial infarction, nephropathy, liver fibrosis, inflammatory bowel disease, and depression [1, 2, 7, 9, 10]. PsA is one of the most common comorbidities associated with psoriasis, reported in 30% of patients with psoriasis globally [2, 7] and about 13.8% of adult patients with psoriasis in Malaysia [5]. The incidence of PsA is higher in patients with moderate-to-severe psoriasis [11] affecting various joints of the body, such as the spine, fingers, knees, toes, and elbow leading to joint pain, inflammation, joint deformity, and impaired functionality [2, 12, 13].

Appropriate treatment modalities are essential for psoriasis management. Mild psoriasis is usually treated with topical medications, including corticosteroids, vitamin D analogs, retinoids, and calcineurin inhibitors [6, 14]. Moderate-to-severe psoriasis is treated with phototherapy and/or systemic therapy (via an oral or parenteral route of drug administration) when topical therapy is not effective [6, 15]. This includes oral systemic agents, and/or biologics [14]. Biologic therapy is strongly recommended for patients with moderate-to-severe psoriasis, PsA, and those unresponsive to conventional treatments [16]. Although the use of biologics in psoriasis is high in the US (37.2%), in Malaysia their use is reported to be quite low (1.1%) [5, 14].

The major challenge with psoriasis treatment is delayed diagnosis, resulting in delayed treatment initiation. Other challenges include lack of adequate treatment, loss of follow-up, and non-adherence to medication [17]. Moreover, social stigmatization toward patients with psoriasis has a negative impact on their quality of life (QoL), affecting their professional life, personal relationships, and mental well-being [18]. Patients are at significantly higher risk of anxiety, depression, sleep deprivation, alcoholism, smoking, and substance misuse, leading to poor medication compliance [19]. Social rejection experienced by these patients at public places (such as beaches, salons, and public showers) is a major concern, particularly in younger adults [20]. Furthermore, absenteeism from work and school due to psoriasis-related pain and itching is responsible for the loss of productivity [18, 20].

Successful management of psoriasis requires early detection followed by appropriate treatment initiation. However, there is a lack of universal psoriasis treatment guidelines and specialists in Malaysia, indicating a lack of prompt diagnosis and appropriate referral, delay in treatment, and poor treatment outcomes. Moreover, inadequate public awareness about psoriasis is responsible for social stigmatization and discrimination. However, in Malaysia, there is a scarcity of patient—perspective studies on psoriasis focusing on the impact of psoriasis on the QoL of patients, the psychological burden of psoriasis, and patient’s attitudes about current treatment modalities.

To address these unmet needs, a patient advisory board meeting was conducted in Malaysia to understand the impact of psoriasis in the everyday life of patients, to uncover the emotional and psychosocial burden faced by patients during their journey with psoriasis, and to identify the perceptions and attitudes of patients toward current psoriasis treatment.

## Materials and methods

A patient advisory board meeting consisting of a chairperson/moderator who is a senior consultant dermatologist and a medical advisor of the Psoriasis Association of Malaysia, and 11 voluntary patients was conducted in person in Malaysia on March 26, 2022. Adult patients of age ≥18 years with mild or moderate-to-severe plaque psoriasis, preferably with concomitant PsA, who were either bio-naïve or bio-experienced and treated in public and/or private hospitals were invited to participate in the meeting.

**Fig 1:**
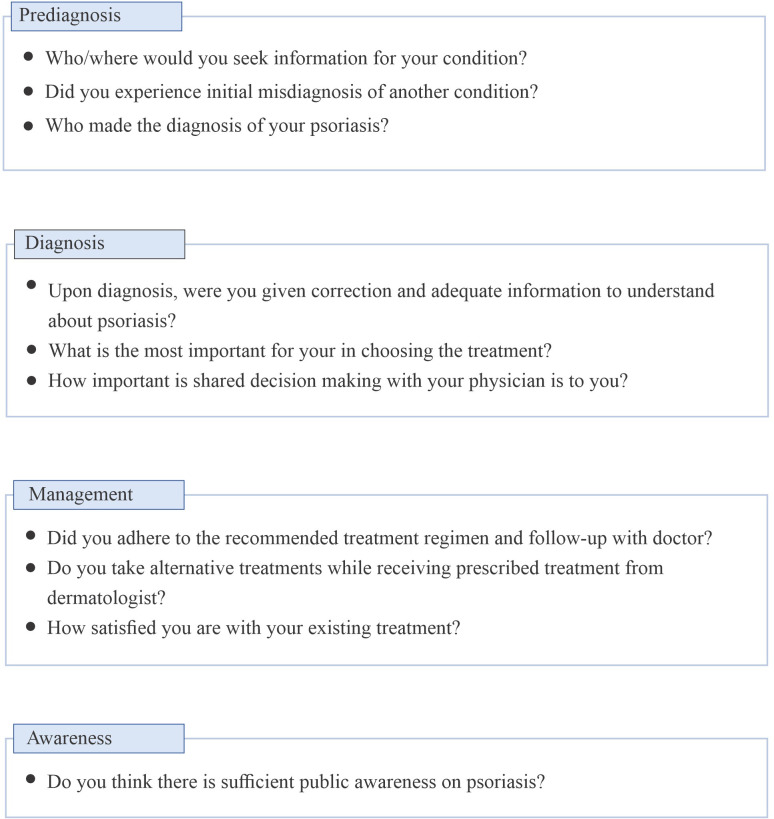
Summary of clinically relevant questions.

A survey questionnaire comprising clinically relevant questions on pre-diagnosis, diagnosis, psoriasis treatment and management, and suggestions for improving the QoL of patients was developed in both English and Malay and then reviewed and approved by the moderator. During the advisory board meeting, the survey questions were displayed on the screen and the patients responded to the questions immediately through the Poll Everywhere (PollEv) app. The clinically relevant questions are summarized in Fig 1.

The objective of the meeting was to discuss the current landscape of psoriasis in Malaysia and patient perspectives in dealing with psoriasis in terms of the appearance of the first symptom and symptom duration, consultation with a physician, presence of other comorbid conditions, and current treatment. Patients were provided with unique participant numbers to facilitate anonymous data collection and to maintain confidentiality. Best practices in the survey design were employed to avoid potential bias. The entire meeting session was video recorded.

Ethics committee clearance meeting was held in Johnson & Johnson Sdn Bhd at Petaling Jaya, Selangor, Malaysia and the approval was obtained from the Janssen Healthcare compliance and legal team for conducting the meeting (Internal event approval code: *BA001-2022-282*). Written informed consent was obtained from each patient before the survey to use the data for publication.

## Results

### Demographic characteristics of patients

A total of 11 patients (five females and six males) of Asian ethnicity participated in the patient advisory board meeting. The mean age of the patients was 46 years (range: 31 to 69 years). The number of years the patients suffered from psoriasis ranged between 8 and 36 years with a mean years of suffering being 24.5 years. Most patients were diagnosed with psoriasis at adult age, with the mean age of psoriasis diagnosis being 21.9 years. Only a few patients were detected at the adolescent age. The details of the patients; their current age, age of psoriasis diagnosis, and year of suffering with psoriasis are presented in Table 1.

**Table 1:** Demographic characteristics of patients.

Serial number of patients	Current age of patients (years)	Age at first psoriasis diagnosis (years)	Year of suffering from psoriasis (years)
1	31	15	16
2	50	33	17
3	45	20–21	>20
4	30	22	8
5	57	25	32
6	41	18	22
7	53	20	33
8	30	19	11
9	51	22	23
10	69	33	36
11	42	13	30

### Initial symptoms of psoriasis and associated challenges

The most reported initial symptom of psoriasis was itching (5/8; 62.5%) associated with a scaly scalp, which the patient initially thought to be dandruff. This was followed by itchy, red plaques in other areas of the body, especially the elbows. One patient described the spreading of psoriasis as:

*“Initially*, *my psoriasis started as dandruff [or dandruff-like lesions] on the scalp*, *followed by red plaques on the elbow*. *However*, *after 4 months*, *the disease spread to my whole body*, *covering 90% of the area with erythrodermic psoriasis*.*”*

Many of the patients believed that the main challenge with psoriasis was their and their family members’ lack of awareness about the initial symptoms of the disease, leading to a delay in physician consultation and treatment initiation. Three patients (27%) reported that it took about two months to two years to consult a doctor and seek treatment for psoriasis after experiencing the first symptom. Typically, the common practice among patients is to gather information from various sources before making an appointment with the doctor. Internet (4/9; 44%), family members (4/9; 44%), and social media (1/9; 11%) were the preferred sources of information among the patients. Moreover, prior to consultation with physicians, the patients even tried various home/natural remedies, including the application of lime juice (2/9; 18%), coconut oil (3/9; 27%), gingelly oil (1/9; 9%), fenugreek seeds (1/9; 9%), neem leaves (1/9; 9%), and turmeric (1/9; 9%). Besides natural remedies, some patients (3/11; 27%) used anti-dandruff shampoo and moisturizers to manage the condition. Moreover, the patients stated that these home remedies were although effective temporarily, later turned out to be ineffective, and mostly led to a flare-up of psoriasis, which necessitated further consultation with the physician. As one patient puts it:

*“I thought it was just dandruff*, *and I did some Google search on the traditional remedy to treat dandruff*. *I tried applying lime juice*, *and that was the moment when I felt that I had made a mistake*.*”*

### Challenges associated with the diagnosis and information on psoriasis

Most patients did not consult a dermatologist in the first instance, which often led to misdiagnosis. Approximately 63% of patients reported initial misdiagnosis of psoriasis and mentioned that more than 3 years were required to obtain a correct diagnosis. Four patients reported misdiagnosis of their condition as other skin diseases, such as eczema, ringworm, and dandruff. In about 90% of patients, psoriasis was diagnosed by a dermatologist rather than by a PCP. One patient underscored the need to consult a doctor to mitigate the delay in treatment initiation:

*“No matter what*, *you must consult the specialist doctor*. *You should not self-diagnose [the skin condition]*. *I know this way [seeking consultation] works for me*. *I will be okay [improvement of my psoriasis] if I take this medicine*. *So*, *you must consult the doctor for the right diagnosis*.*”*

However, one patient was uncertain about the diagnosis and preferred to seek a second opinion and attempted alternative approaches, such as meditation and alternative therapy from traditional healers.

A total of 70% of patients reported receiving correct and adequate information about psoriasis from their physicians upon disease diagnosis, whereas 30% of the patients did not receive adequate information from their treating physician. According to the participating dermatologist, this is not surprising, especially in a government hospital setting, where due to the high volume of patients, physicians often have insufficient time to provide adequate information about the disease, its complications, and appropriate medications.

### Patient expectations regarding psoriasis treatment, adherence to treatment, and dietary factors

Psoriasis is a chronic disease that requires long-term treatment involving topical therapy, phototherapy, systemic therapy, and biologics based on the type of psoriasis, severity, and affected areas. Almost 50% of patients mentioned that they know that psoriasis is not completely curable; however, it can be controlled with proper treatment and follow-up.

When the patients were asked to mention the reason for selecting a treatment or expectation regarding the treatment of psoriasis; overall, 36% reported that the ability to enjoy longer remission with clearer skin was the main reason for choosing a treatment. Other reasons for treatment/expectations from treatment included achieving faster skin clearance (3/11; 27%), following the physician’s recommendation (2/11; 18%), and ease of medication use (1/11; 9%). According to a patient, mostly younger patients preferred having a faster skin clearance (within one to two weeks) than older adult patients.

All patients agreed that joint decision-making with their physicians to understand the feasibility of the treatment regimen and adhering to the treatment are crucial to obtaining optimal outcomes. The majority of patients (9/10; 90%) reported that they accepted their physician-prescribed treatment and did not opt for alternative natural therapies. Only one patient stated that he avoided steroids as they might worsen the condition. One patient mentioned preferring injections over oral medications because in that case, medicines need not be taken regularly for a prolonged period.

There are some reports about certain foods causing flares of psoriasis. Most patients preferred home-cooked meals over processed food to manage their conditions. However, they mentioned that some foods (red meat, sweets, celery juice, etc.) worsened their psoriasis, and it is important to identify those foods and avoid or reduce their consumption:

*“I don’t have any food [even red meat] that affects my condition*. *But I noticed that whenever I ate sweet stuff [food or beverage]*, *e*.*g*., *drink that’s [that is] overly sweet or ice cream like a McDonald’s chocolate sundae cone*, *I realized that my psoriasis would flare up*. *It made the skin very itchy and worse*.*”*

“*I personally felt that celery juice was the trigger for my psoriasis*. *After that [drinking celery juice]*, *it [my psoriasis] flared up and it was bad*.*”*

### Challenges associated with the management of psoriasis

#### Specialist consultation

More than 50% of the participating patients also had PsA, but they preferred to consult only a dermatologist for treatment of both psoriasis and PsA, since consulting dermatologists and rheumatologists was an exhausting and time-consuming process. However, few patients preferred co-management by dermatologist and rheumatologist as they considered this as an ideal treatment strategy:

*“Even though I received biologics from a dermatologist and it’s difficult for me to get an appointment from both a dermatologist and a rheumatologist*, *but for me*, *it’s very important to get the opinion from both specialists…”*

#### Lack of knowledge on medication use and level of patient satisfaction with current treatment

Overall, 73% of patients were highly satisfied with the existing treatment and 27% were somewhat satisfied. Even though all patients reported adherence to the recommended treatment regimen and follow-up appointments with the treating physician, they somewhat faced some challenges in adhering to the treatment. Patients might not be aware of the use of prescribed medications and might get confused with the sequential applications of topical creams. Although sequential topical therapy including the application of topical corticosteroids, calcineurin inhibitors, and salicylic acid in affected areas is beneficial, the patients raised concern about contradictory instructions provided by the doctors and pharmacists at times on the application of the creams. Moreover, one patient mentioned that:

*“Often the physicians assumed that they [patients] understood properly how to use emollient*, *creams*, *and all that*, *but patients go back [home] and they don’t know how to use the cream properly*. *So*, *there must be a way or a toolkit to explain [to] these people how to use these creams properly so that efficacy of the treatment is not lost”*.

These challenges contribute to treatment noncompliance and reduced treatment efficacy since patient satisfaction is an important criterion for treatment adherence.

### Impact of psoriasis on QoL of patients

#### Emotional impact

Almost all patients reported an emotional impact due to psoriasis. Overall, the patients were disappointed with the symptoms of psoriasis. They were shocked (2/11; 18%), sad (1/11; 9%), indecisive (1/11; 9%), and outraged (1/11; 9%), and even blamed their parents for genetic inheritance (1/11; 9%). They experienced low self-esteem (2/11; 18%) and a lack of confidence (3/11; 27%). Patients described diverse emotions (shock, sadness, and outrage) when they first heard about their diagnosis. Moreover, a few patients experienced an emotional breakdown due to the burning sensation on their skin and the associated pain during flare-ups; they mentioned that the pain was extreme and constant. This pain adversely affected their daily activities. One patient shared that he had to clean chairs frequently to remove the skin flakes to avoid embarrassment. In addition, there is an association between flare-ups stress, and poor self-esteem. The impact of psoriasis on the QoL of patients is shown in Table 2.

**Table 2:** Impact of psoriasis on QoL of patients.

Factors	Patient experiences with psoriasis affecting their QoL [Patient serial number]
Emotions	*“My whole body was bleeding at that time [during flare-up]*, *and I could not wear my school uniform*. *So*, *I did not go to school for 2–3 months*.*” ‐ ****[Patient 8]***
	*“I did not have the confidence to go to school during flare-ups*, *so I just studied from home*.*”-* ***[Patient 8]***
	*“Motivation is within*, *courage is most important for psoriasis patients*. *Happiness is a choice*, *but not psoriasis*.*”-* ***[Patient 11]***
Psychosocial impact	*“With just sitting for a while*, *you will need to vacuum again*, *and then*, *[let’s say] if you want to go out and need to sit in someone’s car*, *[oh man*!*] or you need to sit on the black chair*. *Oh*, *man*! *your movement will get disturbed [due to visible flakes]*. *It really affects 100% [of your] emotions*, *it’s not easy*. *It’s not easy handling psoriasis*, *but like what the doctor said it’s up to you*. *It’s you who must get out of there [the feeling of stigmatization]*, *think positively and just be yourself*. *But initially it [the confidence] wasn’t there*, *it takes time*.*”-* ***[Patient 2]***
	*“Every morning*, *I apply the cream to prevent the skin layers [flakes] from falling*. *I worked as a personal driver*, *every time before I leave my workplace*, *I have to vacuum and clean the car so that my boss cannot see the skin flakes*.*”**** ‐ [Patient 4]***
	*“Limitation in dressing [due to psoriasis]*, *in fact*, *I don’t wear sleeveless [dress] or skirts in my entire life because I don’t want to attract the questions [related to psoriasis lesions]*. *Although now I can handle it right*, *I just don’t want to give the explanations*, *so I always cover up*.*”-* ***[Patient 6]***
	*“People don’t understand [the psychosocial impact of psoriasis] when you have meetings and especially if the chair is black*, *you will see all the white flakes there when you get up from the chair*.*” -****[Patient 3]***
	*“I always used to wear a long-sleeve shirt when I was outside*, *even in hot weather*. *This continued until people started asking me whether I live in a European country*.*”-* ***[Patient 4]***
	*“I started to isolate myself*. *My self-esteem got down really*, *and my self-confidence was really low*. *My parents were devastated to see my condition*.*”-* ***[Patient 11]***
Stigma and discrimination in public places	*“I usually explain to the barber about my condition [psoriasis] before that [hair cut] and psoriasis is not transmittable and need not to be scared*. *“Some salons are reluctant to have you as a customer*. *They [the salon employees] don’t even want to touch your hair or cut your hair and they are like [enquired] ‘Oh*, *maybe you want to come some other time*?*’ This is like indirectly saying to you that we [the salon employees] are not welcoming you as a customer*. *This is [a] kind of discrimination*. *This brings down your self-esteem*, *and people not having psoriasis will not understand this problem*.*” ‐ ****[Patient 3]***
	*“Last time when we had outdoor activities*, *e*.*g*., *gym*, *I couldn’t attend any outdoor activities after I got psoriasis because if I wear sports dressing [attire]*, *my skin will be exposed*, *and I feel shy*.*” ‐ ****[Patient 4]*** *“I was standing and holding a bar*, *and one lady was standing next to me*, *and I accidentally hit her*. *Immediately*, *she took a tissue and started to wipe her hand in front of me*. *I felt very sad with this treatment*.*” ‐ ****[Patient 11]***
	*“In a bus*, *a guy standing next to me was checking me out*, *and the moment I lifted my sleeve*, *showing my skin*, *straight away [immediately] he moved away from me*.*”-* ***[Patient 11]***
Stigma and discrimination at work	*“At work*, *we all have key performance indicators to achieve*. *I could manage to meet most of the points*, *but there was one question that showed 30% competency*. *That’s where I failed because I was on medical certificate (MC)*, *[sick leave]*. *My commitment towards work*, *the discrimination [at workplace] was disheartening*, *even though I fought*. *I told [him] I was sick*, *and it was not that I wanted to take MC [sick leave]*. *But at the end the rest [of the questions scored] 100% but because of the MC*, *I lost a bit*.*”-* ***[Patient 2]***
	*“The company once wanted the proof that this is neither AIDs nor any transmittable disease*. *I had to take the trouble to get a doctor’s help to resolve this issue*. *This is embarrassing [for me]”-* ***[Patient 7]***
	*“They [employers] were not giving me an opportunity to prove myself at a corporate level*. *They [employers] said ‘she has not got the ability to run the team and perform*.*’ I proved them wrong because I really put a lot of hard work to achieve the target*. *Normal people without any issues put in 70% of effort whereas we psoriasis patients put 150%*, *going through all the mental distress just to get there [achieve the target] and prove to everybody that you can actually do it*.*”* ***[Patient 11]***
Stigma and discrimination from relatives	*“Personally*, *I’ve experienced discrimination once*. *The first incident was when I attended my distant relative’s open house visit*. *At that time*, *after they [the relatives] saw my skin condition*, *they said that if you were a male*, *we would not allow you to marry a girl who has the same condition*. *Yes*, *this [response] was from a relative but not from a close relative*.*”-* ***[Patient 2]***
	*“People have miscommunication with psoriasis*. *The society does not understand a person’s plight*.*” ‐ ****[Patient 11]***
	*“In family*, *it happens with distant relatives*, *not [with] close relatives*. *They [distant relatives] will ask questions such as*: *“why is your face red”*?* ‐ ****[Patient 1]***

#### Psychosocial impact

Psoriasis leads to a considerable psychosocial impact on patients, affecting their work schedule and QoL. One patient mentioned psoriasis affected the personality of young patients as they were more conscious about their physical appearance. However, the elderly patients were less worried about their appearance and accepted the condition in a better way. Patients reported various instances where psoriasis impacted their work, such as companies requesting the patients to provide proof that psoriasis is not contagious, limitations in dressing appropriately–being very selective in choosing the right clothing for work to reduce skin exposure and avoiding dark-colored clothing, evading the use of black colored chairs in office/meetings to ward off visibly white flakes, and being very selective of activities. Other than its impact on work schedule, missed school days (1/11; 9%) were also reported by a patient. The psychosocial impact of psoriasis described by patients is depicted in Table 2.

#### Social stigma and discrimination

Several patients (5/11; 45%) experienced discrimination or stigmatization behavior in public places, workplaces, and even at home. The QoL was affected to the extent that they had to avoid public pools (n = 1), outdoor activities (n = 2), hair salons (n = 1), and accidental contact with strangers (n = 1). Discrimination was experienced at certain hair salons as the personnel were reluctant to touch or cut their hair. The patients then had to convince the employees of the salons that psoriasis is non-contagious. Providing such explanations negatively impacted the self-esteem of the patients. The impact of social stigma and discrimination on patients is described in Table 2.

At work, human resource personnel were reluctant to deal with employees or interns with psoriasis as they were unaware that psoriasis is non-contagious. One patient had to cover her hair and wear a long-sleeved shirt to avoid stigma. Furthermore, some patients stated that taking frequent medical leave is also challenging. They were discriminated against at work; were not provided with adequate opportunities, and were considered incapable and/or incompetent due to frequent absenteeism from work. Hence, they need to put in extra effort, hard work, courage, and motivation to achieve their targets and prove their competency at work, despite the emotional suffering associated with such discrimination. Stigma and discrimination faced by the patients at work are described in Table 2.

Besides the workplace, a few patients (2/11; 18%) were also discriminated against by their relatives (mostly distant relatives) during family gatherings (Table 2).

### Patient perspective on psoriasis awareness

All patients believed that there is a lack of adequate public awareness of psoriasis and raising appropriate awareness could help to reduce the stigma, discrimination, and misconceptions. One patient even suggested conducting more patient perspective advisory board meetings and educating family members about psoriasis.

*“I urge that [healthcare providers] come up with more campaigns so that more awareness [on psoriasis] can be created*. *It is the time to start an advocacy role to educate others*.*”*

Media can be an excellent means to educate people. However, the patients also mentioned that the media is more interested in discussing different chronic illnesses other than psoriasis since psoriasis is not directly associated with mortality:

*“Psoriasis is not a very interesting theme for the media*.*”*

*“For them [media] there are no fatal cases [due to psoriasis]*, *I don’t think we will get media attention*.*”*

Moreover, the participating dermatologist suggested that continuous awareness programs should be organized to educate the public that psoriasis is not just confined to the skin; it goes beyond the skin and is associated with various comorbidities. The media should be made aware that psoriasis is also a serious disease and a public concern, hence they should play an important role in raising public awareness.

## Discussion

This current qualitative patient perspective study provides insights into the impact of psoriasis on patients and the patient journey. The patient’s views on the treatment and management of psoriasis, the challenges associated with psoriasis, including initial diagnosis, and the effect on QoL were also discussed in this study. The findings from the patient advisory board can aid in understanding the journey of a patient with psoriasis. This in turn can be beneficial in identifying the required support to be provided to these patients. PCPs need to be aware of these unmet needs of the patients and address them appropriately to manage the disease effectively and optimize the QoL of patients.

From this study, it was clear that the patients were aware that psoriasis is not completely curable; however, there is a need to obtain adequate information about psoriasis from the treating physician. This was in line with another study that emphasized the need to inform patients that psoriasis is incurable and can be effectively treated and managed based on the severity and individualized treatment options [19]. The requirement of various therapeutic agents for achieving skin clearance and the erratic nature of psoriasis with varying severity over time, even during treatment, need to be explained well in advance to the patients [19]. Most of the patients in this study reported that their psoriasis was diagnosed by the dermatologist. Similarly, previous studies also mentioned psoriasis diagnosis by dermatologists [21, 22]. Moreover, co-management of psoriasis by a dermatologist and rheumatologist was recommended for better treatment outcomes in patients with psoriasis and PsA [23]. This is in line with the perspective of the participating patients in this study. Patients considered achieving long-term remission status along with clearer skin as the main goal for choosing the psoriasis treatment. Even though clear skin was an important aspect of achieving remission, another patient perspective study reported a weak association between skin clearance and remission [24]. The difference may be due to the different age groups of patients included in the study.

The present study identified the lack of awareness of psoriasis symptoms among PCPs as one of the important factors responsible for the misdiagnosis of psoriasis, leading to delays in specialist referral. This is in accordance with the findings of a previous study, which concluded that educating and creating awareness among physicians from all specialties is warranted for early diagnosis of psoriasis [25]. Moreover, it is also essential to create awareness among dermatologists on psoriasis-associated comorbidities, so that patients suspected of PsA can be referred to a rheumatologist at the earliest to receive appropriate treatment [26]. Furthermore, psoriasis is a chronic disease that requires long-term treatment; therefore, adhering to treatment is crucial for effective disease management and lowering disease severity. Nonetheless, in a study, almost half of the patients with psoriasis were not compliant with the prescribed medication. One of the main reasons for treatment non-adherence is patient dissatisfaction with treatment [27]. In this study, although most of the patients reported that they were highly satisfied with the current treatment, they raised concerns about the lack of proper instructions on the medication use by the physicians and the pharmacists leading to medication non-compliance. Hence, physicians must instruct the patients about the correct use of different creams sequentially to ensure satisfaction and treatment adherence, resulting in improved clinical outcomes.

Psoriasis has a detrimental effect on patients’ QoL limiting their daily routine due to pain, itching/pruritus, and visible, skin lesions leading to embarrassment, anxiety, emotional distress, depression, and poor self-esteem [28]. Additionally, anxiety stemming from visible physical appearance results in social avoidance, limited lifestyle choices, missing workdays, and eventually feelings of isolation and depression [29]. Increased patient communication by dermatologists is essential for psoriasis management to improve the overall QoL of the patients [30]. A cross-sectional study from Northeast Malaysia reported a correlation between symptoms of depression and QoL in patients with psoriasis [31]. In our study, although clinical depression was not reported, self-isolation, absenteeism from school and work, and avoiding inquisitive people have been reported by the patients in line with the previous studies.

Despite therapeutic efforts, psoriasis substantially impacts patient’s psychosocial well-being [30]. Embarrassment and stigmatization at work and in the community can lead to psychological morbidity [32]. Most patients in the present study mentioned facing humiliation and discrimination in the workplace and society because of their skin condition. Similarly, a patient perspective survey conducted on 8,338 patients from 31 countries with moderate-to-severe plaque psoriasis revealed that 84% of patients faced discrimination and even humiliation due to their visible skin symptoms [18]. However, patients can achieve an optimistic outlook despite the stigmatizing behavior of society and exhibit self-acceptance of their skin condition through confidence and support from partners and close relatives [33].

The lack of knowledge on psoriasis, misinformation on the disease etiology, and the promotion of unproven traditional remedies for psoriasis through social media platforms can provide misleading information to the patients. This affects their mental and physical health as well as QoL [34, 35]. Educating the public and healthcare workers, improving their awareness, and creating social acceptance for patients is crucial to mitigating the stigma associated with psoriasis [34]. The physicians and patients who participated in the study also agreed that increased education and awareness among the public and PCPs on psoriasis is vital.

The main strength of this patient perspective study is that it provides insights into the patients and their journey with psoriasis in a real-time scenario. However, this study was limited by its small sample size. In addition, a few patients in this small sample did not respond to certain questions, which might lead to potential bias. Furthermore, all patients who participated in the advisory board meeting were treated for psoriasis in a single hospital. Therefore, the insights gained from the patient advisory board may not be generalized to the entire country. Evaluating this matter in a more diverse population involving a large sample size from diverse clinics could provide corroborative evidence for future investigations.

## Conclusion

Psoriasis is a chronic disease requiring long-term treatment. Early diagnosis followed by appropriate treatment modalities is essential for effective psoriasis management. Overall, this patient perspective study demonstrated a lack of appropriate knowledge of the disease symptoms among the PCPs and patients, leading to an increased chance of misdiagnosis and delayed treatment initiation. This subsequently resulted in aggravation of the condition and poor treatment outcomes. Moreover, the study highlighted that the psychological and emotional burden on patients with psoriasis stemming from the lack of awareness of society about psoriasis leads to stigmatization and discrimination. This has a significant negative impact on patients’ personal, professional, and social lives, which worsens their QoL. The patients agreed that public awareness about psoriasis is lacking considerably. The findings of the study suggested that raising widespread public awareness about psoriasis is essential to alleviate social stigma/discrimination and create social acceptance of these patients. Additionally, educating PCPs as well as the patients about psoriasis symptoms is necessary for early diagnosis, specialist referral, and timely treatment initiation for effective management of the disease.
